# Adipose tissue protein profiling: modulation by vitamin D receptor

**DOI:** 10.3389/fnut.2026.1715659

**Published:** 2026-03-10

**Authors:** Ding Ding, Chengmei Zhang, Tiantian Xia, Yu Chen, Yang Liu, Yan Lou, Juan Kong

**Affiliations:** 1 Department of Clinical Nutrition, Shengjing Hospital of China Medical University, Shenyang, China; 2 School of Public Health, Zhejiang Chinese Medical University, Hangzhou, China; 3 Department of Student Affairs, The First Affiliated Hospital of Jinzhou Medical University, Jinzhou, China; 4 Department of Orthopedics, The Second Affiliated Hospital of Liaoning University of Traditional Chinese Medicine, Shenyang, China; 5 Department of Fundamental Sciences, China Medical University, Shenyang, China

**Keywords:** adipose tissue, bioinformatics analysis, PRM verification analysis, succinylation, TMT proteomics, vitamin D receptor

## Abstract

**Background:**

Little is known regarding the expression patterns of adipose tissue proteins in the context of vitamin D deficiency and whether these expression patterns have adverse effects on fat-related diseases.

**Methods:**

This study compares vitamin D receptor-knockout (VDRKO) and wild-type (WT) mice to determine whether the VDRKO affects the adipose tissue landscape. High-throughput proteomic technology and parallel reaction monitoring-based targeted proteomics were utilized to determine and verify protein level changes.

**Results:**

Integrated proteomic and succinylomic analyses revealed that VDR deletion profoundly reprograms the adipose tissue molecular landscape. We identified 572 differentially expressed proteins and 313 differentially succinylated proteins. In VDRKO mice, protein levels involved in biological regulation, metabolic processes, ribosome, and endoplasmic reticulum protein processing pathways were upregulated. Conversely, proteins serving as negative regulators were enriched in pathways such as complement and coagulation cascades and protein digestion and absorption. Notably, ribosomal proteins (e.g., pancreatic alpha-amylase: Amy2 and proliferation-associated protein 2G4:Pa2g4) were significantly upregulated, while collagen proteins (e.g., Col24a1, Col6a4) were identified as key downregulated regulators in the protein digestion and absorption pathway. Succinylome analysis further indicated extensive succinylation modifications on proteins associated with energy metabolism pathways, including alanine, aspartate and glutamate metabolism, and arginine biosynthesis. These modifications were prominent not only in mitochondria but also in the cytoplasm, suggesting a broad regulatory role for succinylation beyond mitochondrial metabolism in the VDR-deficient state.

**Conclusion:**

This integrated multi-omics study provides the first comprehensive proteomic and succinylomic profile of VDRKO adipose tissue, revealing succinylation as a novel regulatory layer in energy metabolism. Our findings advance the understanding of vitamin D signaling in adipose biology and highlight potential therapeutic targets for metabolic disorders.

## Graphical abstract

**Figure:**
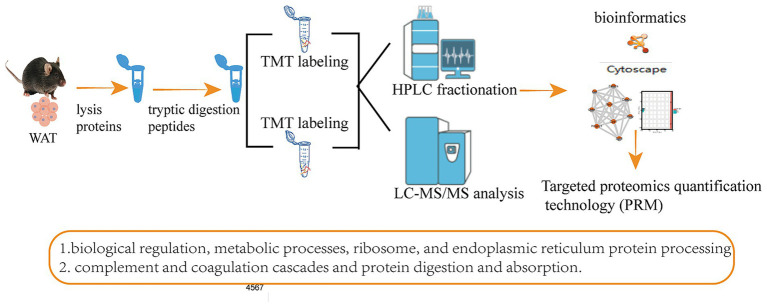

## 1 Background

Vitamin D deficiency is a global health concern, closely associated with obesity and metabolic syndromes such as type 2 diabetes (1–3). The vitamin D receptor (VDR), a nuclear transcription factor abundantly expressed in adipose tissue, mediates both the classical genomic actions of vitamin D on calcium homeostasis and its non-classical effects on metabolic regulation (4–7). Evidence from VDR knockout (VDRKO) mouse models demonstrates that VDR signaling directly influences adipose biology, as these animals display markedly reduced adiposity and dysregulated glucose metabolism (8–10). Despite these phenotypic observations, the specific molecular mechanisms whereby VDR orchestrates adipose tissue proteome remodeling to control metabolic homeostasis remain poorly defined (11, 12).

Adipose tissue functions as an active endocrine organ that coordinates systemic metabolism through secreted factors and dynamic cellular responses (13, 14). Post-translational modifications (PTMs) provide a critical regulatory layer for rapid fine-tuning protein activity in response to metabolic cues (15–17). Lysine succinylation has recently emerged as a key metabolite-sensitive PTM that directly couples cellular succinyl-CoA availability—a central TCA cycle intermediate—to functional modulation of metabolic enzymes and signaling pathways (18). The succinylome is now recognized as an important contributor to metabolic reprogramming across tissues, including adipose, yet its regulation in this context remains largely unexplored (19).

Two fundamental research gaps are evident: First, a comprehensive mapping of VDR-dependent alterations in the adipose tissue proteome is currently lacking. Second, the potential role of VDR in directing protein succinylation—a sensitive gauge of metabolic flux—within adipose tissue remains completely unknown. We hypothesize that VDR ablation drives extensive reprogramming of both the proteome and succinylome in adipose tissue, resulting in metabolic pathway alterations that underlie the observed phenotypic changes in VDRKO mice (20–23).

To address these questions, we leveraged the VDRKO mouse model to investigate cell-autonomous functions of VDR in adipose tissue under vitamin D-deficient conditions. Through integrated proteomic and succinylomic profiling, we systematically mapped VDR-dependent changes in protein expression and succinylation patterns. Our findings establish a novel mechanistic framework for understanding how vitamin D signaling coordinates transcriptional and post-translational regulatory networks to shape adipose metabolism, revealing potential therapeutic strategies for adipose-related metabolic diseases.

## 2 Materials and methods

### 2.1 Animals

Six aged 8–10 weeks (weight: 32–34 g) male mice, were obtained from science and technology innovation center of Shengjing Hospital of China Medical University (Shenyang, China) and randomly divided into two groups (3 mice/group): the WT and VDRKO group. This animal experiment was conducted by the China Medical University Committee on the Care and Use of Animals (approval number: 2017PS267K). And confirming that all experiments were performed in accordance with relevant guidelines and regulations. The specific pathogen-free (SPF) WT and VDRKO mice were generated via the breeding of heterozygous mice housed in science and technology innovation center of Shengjing Hospital of China Medical University (on a C57BL/6 background) previously described (24). Wild-type littermates were used as controls in the experiments. All mice were fed a standard rodent chow diet with detailed nutritional composition (as-fed basis, 90% dry matter): crude protein 198.3 g/kg, crude fat 59 g/kg, crude fiber 42 g/kg, crude ash 63 g/kg, calcium 10.3 g/kg, total phosphorus 8.4 g/kg, potassium 8.6 g/kg, sodium 2.0 g/kg, and vitamin D 4.99 × 10^3^ IU/kg and tap water were provided and maintained at a temperature of 22–25 °C under 12-h light/12-h dark cycle (lights turned on at 6:00 a.m.) daily. Humidity is controlled at 50% ± 10%. Standard environmental enrichment—nestlets and a transparent plastic shelter—was provided in every cage and changed weekly. Genotyping procedures were carried out by PCR using mouse tail genomic DNA^241111^. Mice were anesthetized by isoflurane, and the adipose tissues were harvested and snap-frozen for proteomics and succinylome analysis. Specifically, we first anesthetize the mice with isoflurane at a concentration of 2–3% and a flow rate of 0.2–0.3 L/min and then collect blood samples for storage. Afterward, while the mice are still under anesthesia, we perform euthanasia by exsanguination through the abdominal aorta until they meet the criteria for humane euthanasia, after which we collect samples of abdominal white adipose tissues. And all efforts were made to ensure the animals’ well-being throughout the study. We included only male mice to minimize biological variability arising from oestrous-cycle-dependent fluctuations in sex hormones, which are known to modulate adipose-tissue metabolism and mitochondrial function. This design increases internal validity in our exploratory mechanistic study, but precludes inference of sex-specific effects; future studies must therefore incorporate both sexes to establish generalizability.

## 3 Experimental procedures

### 3.1 Protein extraction and trypsin digestion

The adipose tissue sample was pulverized into cell powder using liquid nitrogen and then transferred to a 5-mL tube. A high-intensity ultrasonic processor was used to sonicate the cell powder three times on ice after it had been treated with 4 mL of lysis buffer. Centrifugation at 12,000 g for 10 min at 4 °C was used to remove the tissue debris. Following the manufacturer’s instructions, the supernatant was collected, and BCA assay was used to test the protein content. For digestion, 5 mM dithiothreitol was added for 60 min at 37 °C for reduction and 11 mM iodoacetamide was added for 45 and then alkylated at 23 ± 2 °C in the dark. Then added 100 mM TEAB to dilute protein sample, and the urea concentration was less than 2 M. Finally, the trypsin-to-protein mass ratio was 1:50 for the first digestion which was performed overnight, followed by a 1:100 trypsin-to-protein mass ratio for the second 4-h digestion.

### 3.2 Tandem mass tag labeling

Firstly, tryptic peptides were dissolved in 0.5 M TEAB. Each channel of peptides was labeled with their respective tandem mass tag (TMT) reagent (this process based on manufacturer’s protocol, Thermo Scientific) and then incubated for 2 h at room temperature. Quality control (QC): After TMT labeling, an aliquot of each multiplex was pooled to create a “bridge” channel that was injected every five LC-MS/MS runs. This bridge was used to monitor median peptide ion intensity, missed-cleavage rate and retention-time drift; runs were repeated whenever the bridge intensity dropped >15% or retention-time drift exceeded 0.5 min. Secondly, samples were quenched by adding 5% hydroxylamine. The pooled samples were then desalted with Strata X C18 SPE column (Phenomenex) and dried by vacuum centrifugation. Technical replicates: Each biological sample was trypsin-digested and labeled in duplicate with two different TMT-10-plex channels. Both technical replicates were measured in the same LC-MS/MS session; the reporter-ion intensities of the two channels were averaged before downstream analysis. Batch normalization: Samples were randomized across two TMT 10-plex sets. After acquisition, reporter-ion intensities were first log2-transformed and then median-center normalized within each TMT set. The effectiveness of correction was verified by inspecting the first two principal components before and after ComBat; no residual clustering by batch was observed. For TMT of proteins/post-translational modifications (PTMs), we first required: a minimum of two unique peptides per protein (or one unique modified peptide per PTM site); a valid TMT intensity in ≥2/3 of biological replicates in at least one group. Statistical significance was assessed by two-sided Student’s *t*-test (or Welch *t*-test when variances were unequal) with Benjamini–Hochberg correction. We used an adjusted *p*-value (*q*-value) <0.05 as the sole significance threshold. Proteins/PTMs with *q* < 0.05 were considered statistically differentially abundant, regardless of their fold change.

### 3.3 Succinylation modification enrichment and LC-MS/MS analysis

Tryptic peptides dissolved in NETN buffer (with 100 mM NaCl, 1 mM EDTA, 50 mM Tris-HCl, 0.5% NP-40, pH 8.0) were incubated with pre-cleaning antibody beads (PTM-402, PTM Biolabs). In short, the beads were then washed with buffer and H_2_O. Bound peptides were eluted from the beads in 0.1% trifluoroacetic acid. Then the eluted fractions were combined and vacuum dried. For liquid chromatography-mass spectrometry analysis (LC-MS/MS), the peptides were desalted using C18 ZipTips (Millipore), according to the manufacturer’s instructions.

### 3.4 Database search

In a word, the mascot search engine (v 2.3) was used to handle MS/MS data. The mouse SwissProt database (16,991 entries) was conducted to match Tandem mass spectra. Using trypsin/P as the cutting enzyme allows up to 2 missing cuts, and for the succinylated proteome, the maximum missing cuts is set to 4. Peptide and protein identifications were filtered at a false discovery rate (FDR) of 1% estimated by the target-decoy approach. Differentially expressed genes were defined by ≥|2.0|-fold change, FDR-adjusted *p* < 0.05, and required to be detected in ≥3 biological replicates per group; genes with low reproducibility (present in <3 replicates) were excluded to reduce the impact of biological variability.

## 4 Bioinformatics methods

The data that support the findings of this study are available on request from the corresponding author. The data is not publicly available due to privacy or ethical restrictions.

### 4.1 Gene Ontology annotation

Gene Ontology (GO) is a conventional bioinformatics tool. Particularly, the objectives of this project are as follows: (1) establish and enhance a controlled vocabulary of gene and gene product attributes; (2) provide annotation gene products and distribute annotation data; and (3) offer user-friendly tools access comprehensive project data. The GO analysis covered three parts: (1) cellular component (CC), molecular function (MF) as well as biological process (BP). The UniProt-GOA database^1^ was utilized to obtain the GO annotation proteome. Proteins were then classified by GO annotation based on three categories: BP, CC, and MF. GO enrichment analysis was performed with Fisher’s exact test, and terms with FDR <1% were considered significant. The *p*-value was adjusted to <0.05.

### 4.2 Kyoto Encyclopedia of Genes and Genomes pathway annotation

Analysis of enrichment was performed using the Kyoto Encyclopedia of Genes and Genomes (KEGG) integrates existing knowledge on molecular interaction networks. These databases constitute distinct networks known as the “protein network” and the “chemical universe.” The KEGG pathways mainly involved metabolism, genetic information processing, environmental information processing, cellular processes, rat diseases, and drug development. KEGG enrichment analysis was performed with Fisher’s exact test, and terms with FDR <1% were considered significant. The *p*-value was adjusted to <0.05. KEGG online service tools KAAS^2^ and KEGG mapper^3^ were used.

### 4.3 Subcellular localization

We utilized WoLF PSORT, a subcellular localization prediction software. Wolfpsort is an updated version of PSORT/PSORT II that is specifically designed for the predicting eukaryotic sequences.

### 4.4 Enrichment-based clustering

Hierarchical clustering is based on the functional classifications of DEPs (including GO, domains, pathways, and complexes). The resulting filtered *p*-value matrix was transformed using the equation *x* = −log10 (*p*-value). Finally, the *x*-values were *z*-transformed for each functional category. The *z*-scores were then subjected to one-way hierarchical clustering (using Euclidean distance and average linkage clustering) in genesis. The membership of clusters was visualized using a heat map generated by the “heatmap.2” function from the R-package “gplots.”

### 4.5 Pathway enrichment analysis

Gene set enrichment analysis (GSEA) was employed to assess pathway enrichment. GSEA evaluates and determines whether predefined gene sets, showing cumulative statistically significant changes in gene expression, was classified into specific phenotypes. The enrichment score (ES) in GSEA was calculated by ranking the proteins from the most to the least significant associated with the two groups and then analyzing how the proteins within each gene set were distributed across the ranked list.

### 4.6 Protein–protein interaction network

We used STRING database version 11.0^4^ for protein–protein interactions. STRING employs a confidence score, and we retrieved interactions with a confidence score ≥ 0.7 (indicating high confidence). The interaction network from STRING was visualized via the R package “network D3.”

### 4.7 Parallel reaction monitoring to validate proteomic analyses

To process the resulting MS data, we employed Skyline (v.20.1). The peptides were configured as follows: the enzyme was specified as trypsin (KR/P), allowing for a maximum of two missed cleavage. The peptide length was set to 7–25 amino acids. Fixed modifications included carbamidomethylation on cysteine, and acetylation at the protein N-terminus and oxidation on Met were specified as variable modifications. The variable modifications used were carbamidomethyl on Cys and oxidation on Met. Finally, the maximum number of variable modifications was limited to three. Transition settings were defined as precursor charges were set to two or three, ion charges were set to one or two, and ion types included b, y, and p ions. The number of product ions ranged from 3 to the last ion, with an ion-matching tolerance of 0.02 Da. Peptide ions for parallel reaction monitoring (PRM) monitoring were chosen from the discovery cohort after retaining only those with Gaussian-shaped peaks, reproducible MS/MS matches across injections, and S/N ≥10. Candidates exhibiting inter-run CVs below 15% among three technical replicates were subsequently used for quantification. The process of gradually narrowing down from quantitative proteins: Step 1: Retain proteins with a TMT fold change ≥2.0 and FDR <1%. Step 2: Eliminate those without protectotypic peptides or with predicted responses that are too low. Step 3: Prioritize 15 candidates that are key nodes in disease pathways (top 20% in IPA topology score) and supported by literature as potential biomarkers; ultimately, select 5 qualified protein molecules based on the cost of synthetic peptides and PRM throughput.

### 4.8 Statistical analysis

Proteomic profiling was carried out with three independent biological replicates. Correlation between TMT and PRM quantification for the five selected DEPs was analysed in GraphPad Prism 8.0.2 (263).

## 5 Results

### 5.1 Quantitative profiling reveals 572 altered proteins and 466 succinylation sites in VDRKO adipose tissue

**Figure 1:**
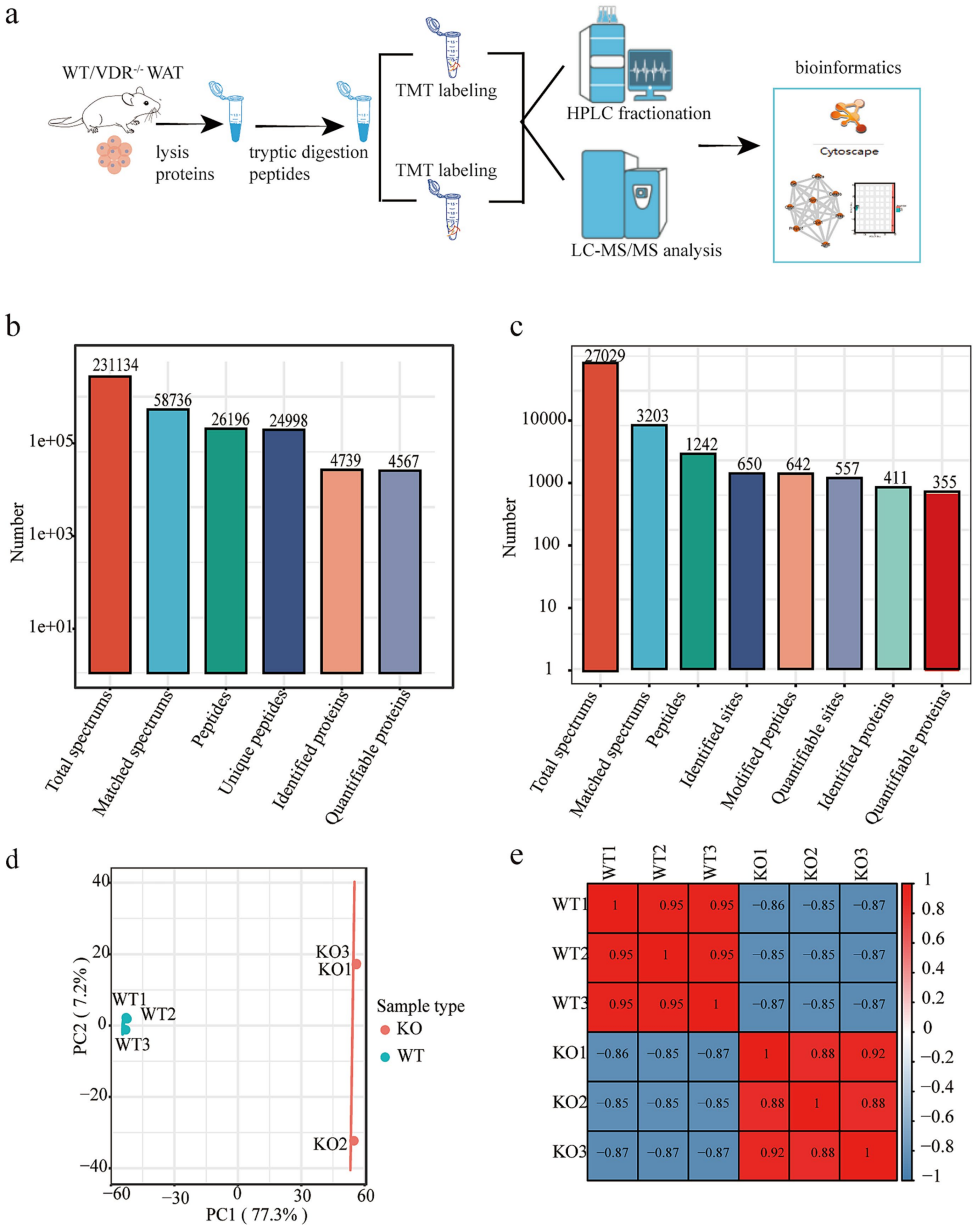
Profiling analysis of the proteome and succinylome of adipose tissue from wild-type (WT) and vitamin D receptor knockout (VDRKO) mice. **(a)** Experimental design for proteomic and succinylomic analyses of adipose tissues from WT and VDRKO mice. **(b,c)** Overview of protein identification of the proteome and succinylome. (1) Total spectra: the number of total spectra of secondary spectra produced by mass spectrometry. (2) Matched spectra: the number of effective spectra and the number of spectra that match the theoretical secondary spectra. (3) Peptides: the number of identified peptides and the number of peptide sequences resolved by matching results. (4) Unique peptides: the number of unique peptides identified and the number of unique peptide sequences resolved by matching results; (5) The number of identified proteins is the number of proteins resolved by specific peptides. (6) Quantifiable proteins: The number of proteins quantified by specific peptides. **(d)** Principal component analysis (PCA) comparing VDRKO samples (red) with WT samples (blue). **(e)** Heatmap of pairwise Pearson correlation coefficients between WT and VDRKO adipose tissue samples. A Pearson correlation coefficient close to −1 indicates a negative correlation, a value close to 1 indicates a positive correlation, and a value close lose to 0 indicates no correlation.

To clarify the regulation of metabolic disorder-induced inflammation in VDRKO mice, six adipose tissue samples were collected from WT and VDRKO mice. For quantitative proteomic analysis, TMT labeling, HPLC, and liquid chromatography-tandem mass spectrometry was conducted. A schematic of the workflow is shown in Figure 1a. Therefore, this study offers an entire summary of adipose tissue at the multiomics level. The comprehensive analysis of lysine succinylation and the proteomic profiling of mouse adipose tissue revealed that of the 4,739 proteins identified, 4,567 were successfully quantified (Figure 1b). A total of 1,242 peptides were identified as succinylated, and 355 quantifiable proteins across 411 identified proteins were quantified by profiling adipose tissue data from WT and VDRKO mice. Our comprehensive proteomic and succinylomic profiling of mouse adipose tissue yielded robust datasets with high quantitative coverage. As summarized in Figure 1b, the core proteomic analysis generated 231,134 total spectra, with 58,726 matched spectra (reflecting data utilization efficiency). We identified 26,198 peptides, including 25,958 unique peptides, demonstrating high protein identification specificity. From these data, 4,739 proteins were identified, with 4,567 successfully quantified, representing excellent quantitative coverage of the adipose tissue proteome. Parallel succinylome analysis (Figure 1c) revealed extensive lysine succinylation modifications, with 72,029 total spectra acquired and 3,203 matched spectra. We identified 1,242 succinylated peptides corresponding to 650 modification sites, of which 642 were confidently localized modified sites and 557 were quantifiable sites. This included 411 identified succinylated peptides, with 355 quantifiable peptides representing 355 quantifiable succinylated proteins from adipose tissue of both WT and VDRKO mice. Prior to a series of sample quality control (QC) evaluations and standardization processes, principal component analyses (PCAs) of the proteomics samples were performed. PCA revealed that the high separation between each group had nonparallel enrichment features (Figure 1d). The heatmap of Pearson’s correlation coefficient results was consistent with that of the PCA. Replicates were repeated well in the WT and VDRKO cells (Figure 1e).

**Figure 2:**
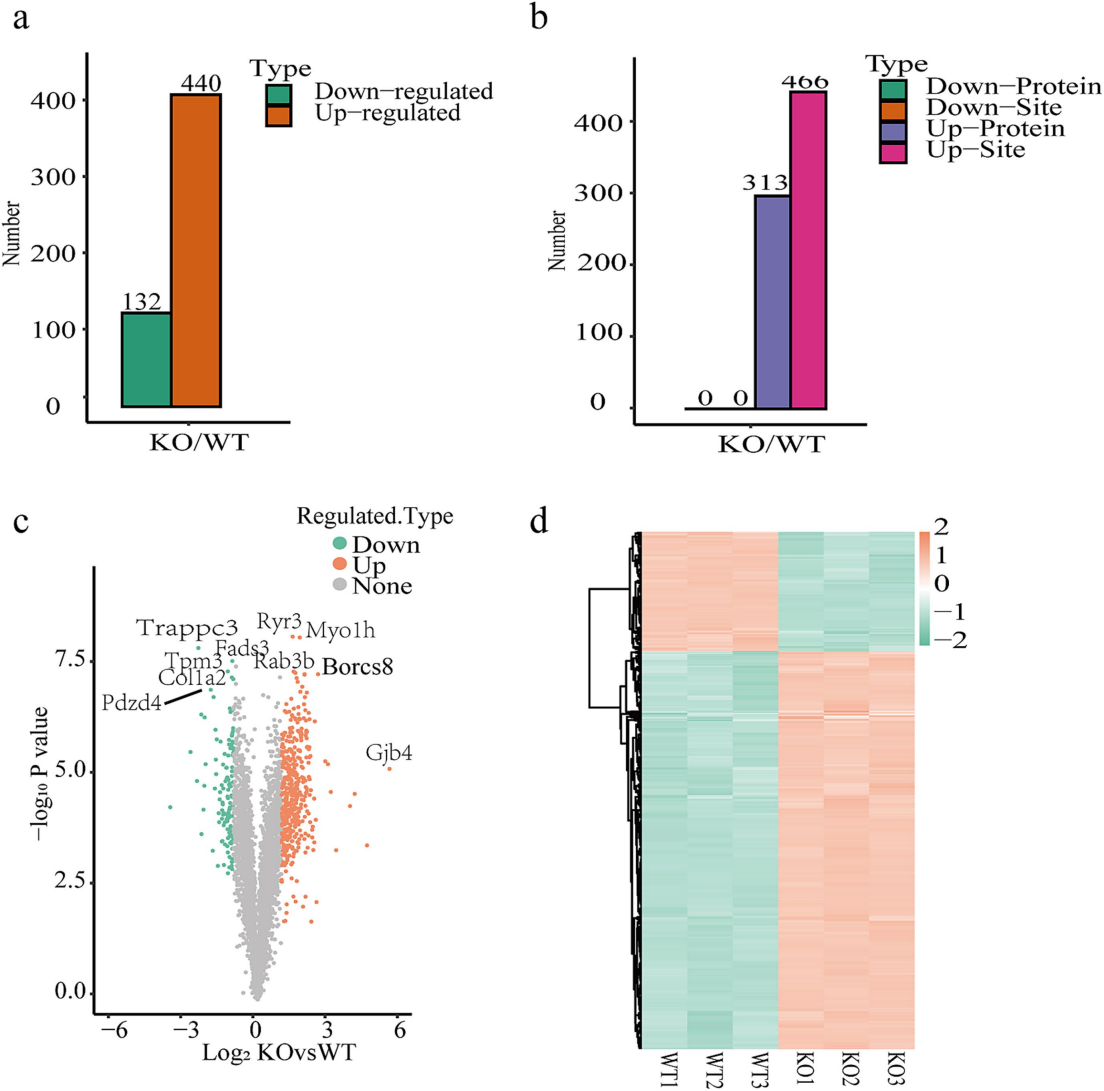
Quantitative proteomics data of WT and VDRKO adipose tissues. Graphs show the number of differentially expressed proteins (DEPs) for **(a)** proteomics as well as **(b)** succinylated proteins and sites (the data from proteomics were used for normalization) [Fold-change threshold: absolute value ≥2 (|log_2_FC| ≥1), Fold-change ≥2.0 up-regulation/ ≤0.5 down-regulation; statistical significance threshold: adjusted *p*-value <0.05]. Note that the red and green plots represent the proteins significantly up- and downregulated in the proteome, respectively. The purple and pink plots indicate the significantly upregulated proteins and succinylation sites. **(c)**. Volcano plot showing DEPs with a fold change (FC) ≥2 and a *p*-value ≤0.05. Orange indicates upregulated proteins, whereas green represents downregulated proteins. **(d)** Heatmap differences at the proteomic level according to hierarchical clustering analysis of adipose tissue from WT and VDRKO mice. The data represent the means ± SEMs (*n* = 3 per group).

Proteomic analysis identified 572 differentially expressed proteins with a 1% FDR. The results showed 440 proteins were upregulated in VDRKO adipose tissue, and 132 were downregulated (Figure 2a), compared to the control group. Succinylome analysis revealed 313 upregulated proteins. In addition, 466 succinylated sites corresponding to 313 succinylated proteins were recognized with a good site localization score, providing a chance to reveal the interaction between the succinylome and the full proteome (Figure 2b). The overall distribution of protein expression differences was visualized via volcano plots, and heatmaps are shown in Figures 2c,d, respectively.

### 5.2 Systematic functional annotation reveals enrichment of metabolic and cellular processes in VDRKO adipose tissue proteome and succinylome

**Figure 3:**
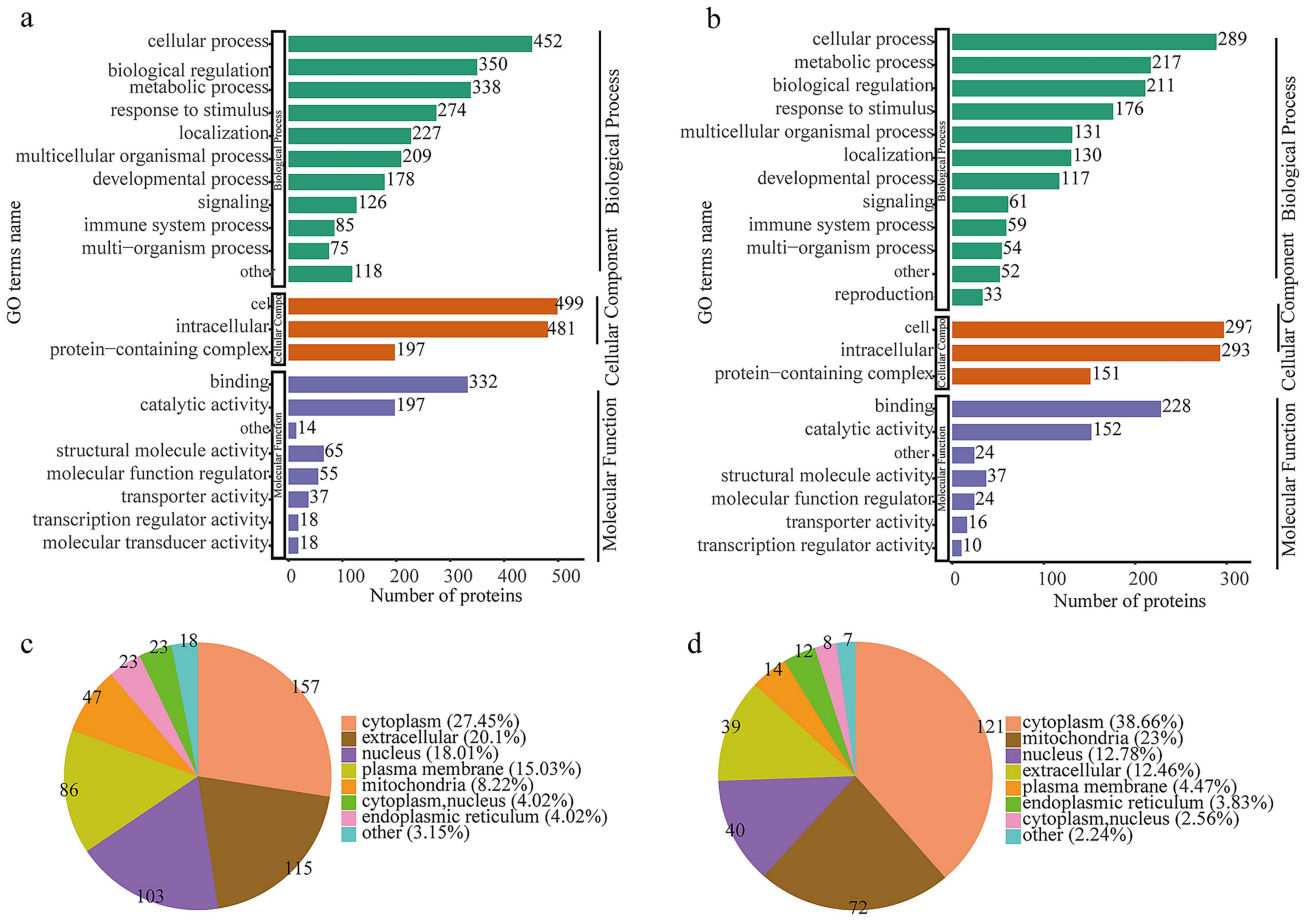
Classification of the proteome and succinylated protein features derived from adipose tissue samples from WT and VDRKO mice. **(a)** Gene Ontology (GO) term characterization of the identified proteomic proteins based on biological process (BP), cellular component (CC), and molecular function (MF) categories. **(b)** Classification of the succinylated proteins. The signatures of the subcellular structure of **(c)** proteomics and **(d)** succinylated proteins.

To comprehensively characterize the biological properties of altered proteins in VDRKO adipose tissue, we conducted detailed Gene Ontology (GO) annotation analysis of both the proteome and succinylome. As shown in Figure 3a, GO analysis of the proteome revealed significant enrichment in fundamental biological processes including cellular process (~500 proteins), metabolic process (~450 proteins), and biological regulation (~380 proteins). Response to stimulus (~300 proteins) and localization (~280 proteins) were also prominently represented. For cellular components, the predominant terms were cell (~322 proteins), cellular component (~197 proteins), and organelle (~191 proteins). Molecular function analysis identified binding (~300 proteins), catalytic activity (~280 proteins), and structural molecule activity (~150 proteins) as the most enriched categories. These patterns indicate that VDR deletion broadly impacts core cellular physiology and metabolic pathways in adipose tissue, particularly affecting proteins involved in cell proliferation, differentiation, signal transduction, and metabolic regulation. Parallel analysis of the succinylome (Figure 3b) showed similar but distinct patterns, with cellular process (~360 proteins), metabolic process (~310 proteins), and biological regulation (~211 proteins) remaining the dominant biological process terms. Notably, developmental process (~117 proteins) and immune system process (~94 proteins) showed lower representation. Cellular component terms included cell (~292 proteins), cellular component (~151 proteins), and organelle (~143 proteins), while molecular functions were dominated by binding (~258 proteins) and catalytic activity (~152 proteins). The succinylome profile suggests that succinylation modifications preferentially target metabolic enzymes and structural proteins rather than broad signaling regulators, indicating a specific role in modulating metabolic pathway activity. Subcellular localization analysis using WoLF PSORT provided additional mechanistic insights (Figures 3c,d). For the total proteome, 27.45, 20.1, and 18.01% of annotated proteins were localized to the cytoplasm, extracellular space, and nucleus, respectively, consistent with the secretory nature of adipokines and cytoplasmic metabolic proteins. Strikingly, succinylated proteins showed distinct localization patterns with 38.66% in cytoplasm, 23% in mitochondria, 12.78% in nucleus, and 12.46% in extracellular space. The pronounced mitochondrial enrichment of succinylated proteins, combined with the metabolic process enrichment in GO analysis, provides direct evidence that succinylation modifications primarily regulate energy metabolic pathways within mitochondria, representing a key molecular mechanism through which VDR regulates adipose tissue metabolism.

### 5.3 Pathway enrichment analyses reveal upregulated ribosome and ER processing pathways and downregulated complement cascades in VDRKO adipose tissue

**Figure 4:**
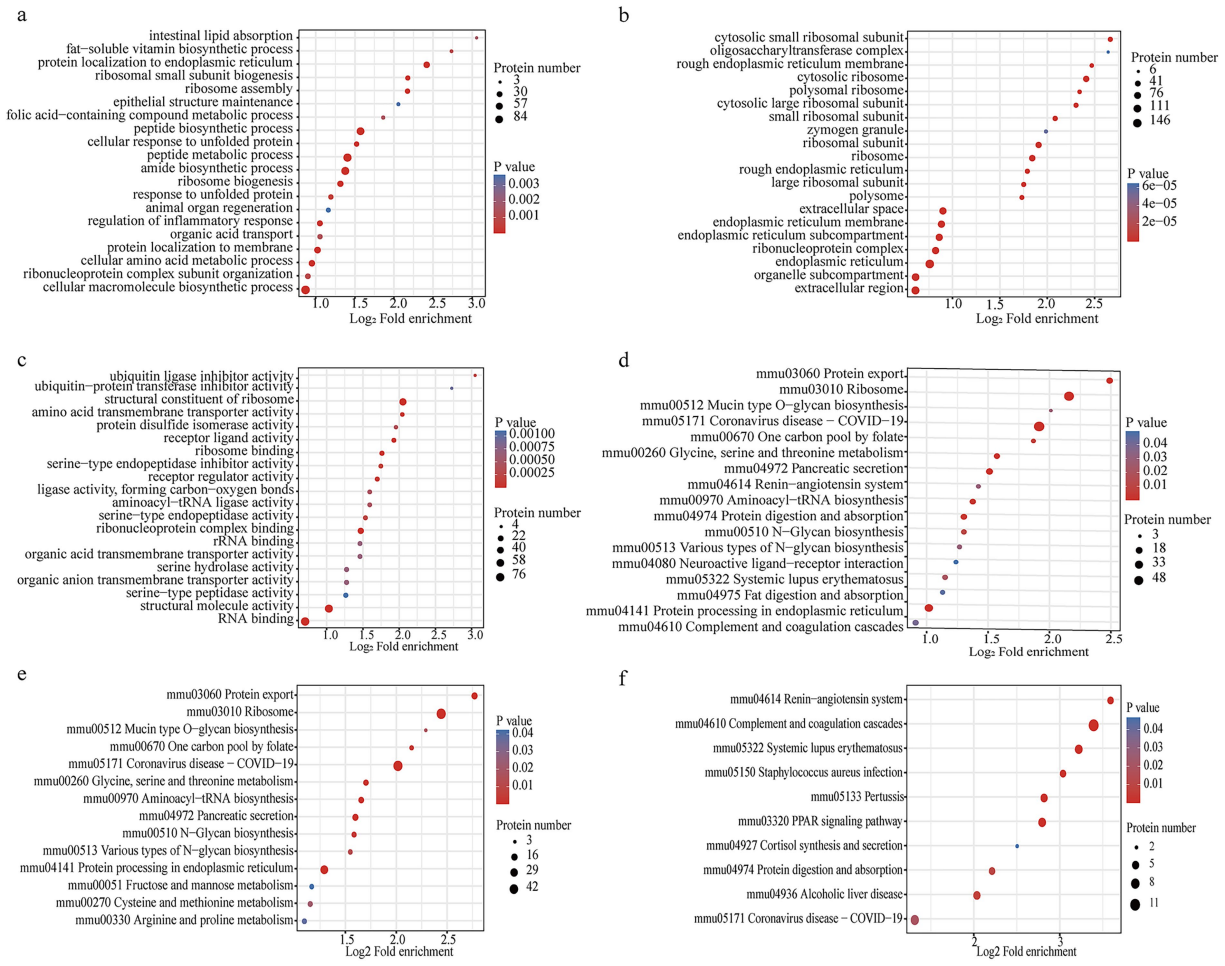
Proteomic enrichment analysis of the WT and VDRKO samples. **(a–c)** The bubble of Gene Ontology (GO) enrichment was grouped into biological process (BP), cellular component (CC), and molecular function (MF) categories. **(d)** Kyoto Encyclopedia of Genes and Genomes (KEGG) pathway analysis of proteomics data. **(e)** KEGG pathway enrichment of upregulated differentially expressed proteins. **(f)** KEGG pathway enrichment of downregulated differentially expressed proteins. Notably, the scale bar with color on the right and the size of the nodes were positively related to their protein numbers.

We then conducted GO and KEGG enrichment pathway analyses of the differentially expressed proteins (DEPs) to gain a complete understanding of the different types of identified proteins and their major biochemical functions. The analysis revealed that ribosome biogenesis, response to unfolded proteins, amide biosynthetic processes and regulation of the inflammatory response were significant pathways involved in biological processes (BPs) (Figure 4a). GO terms for CCs revealed that the DEPs were drastically enriched in the endoplasmic reticulum, ribosome, and rough endoplasmic reticulum membranes (Figure 4b). VDRKO resulted in proteomic changes with respect to different molecular functions (MFs), including constituents of ribosome structure, rRNA binding, and structural molecule activity (Figure 4c). The results of the KEGG pathway analysis revealed that terms such as “ribosome,” “coronavirus disease-COVID-19,” and “protein processing in the endoplasmic reticulum” were distinctly enriched (Figure 4d). Further pathway analysis revealed that proteins whose expression was positively correlated were related mainly to “ribosome,” “coronavirus disease—COVID-19,” and “protein processing in the endoplasmic reticulum pathways” (Figure 4e), whereas proteins whose expression was negatively correlated were involved in complement and coagulation cascades, systemic lupus erythematosus, and protein digestion and absorption pathways (Figure 4f).

**Figure 5:**
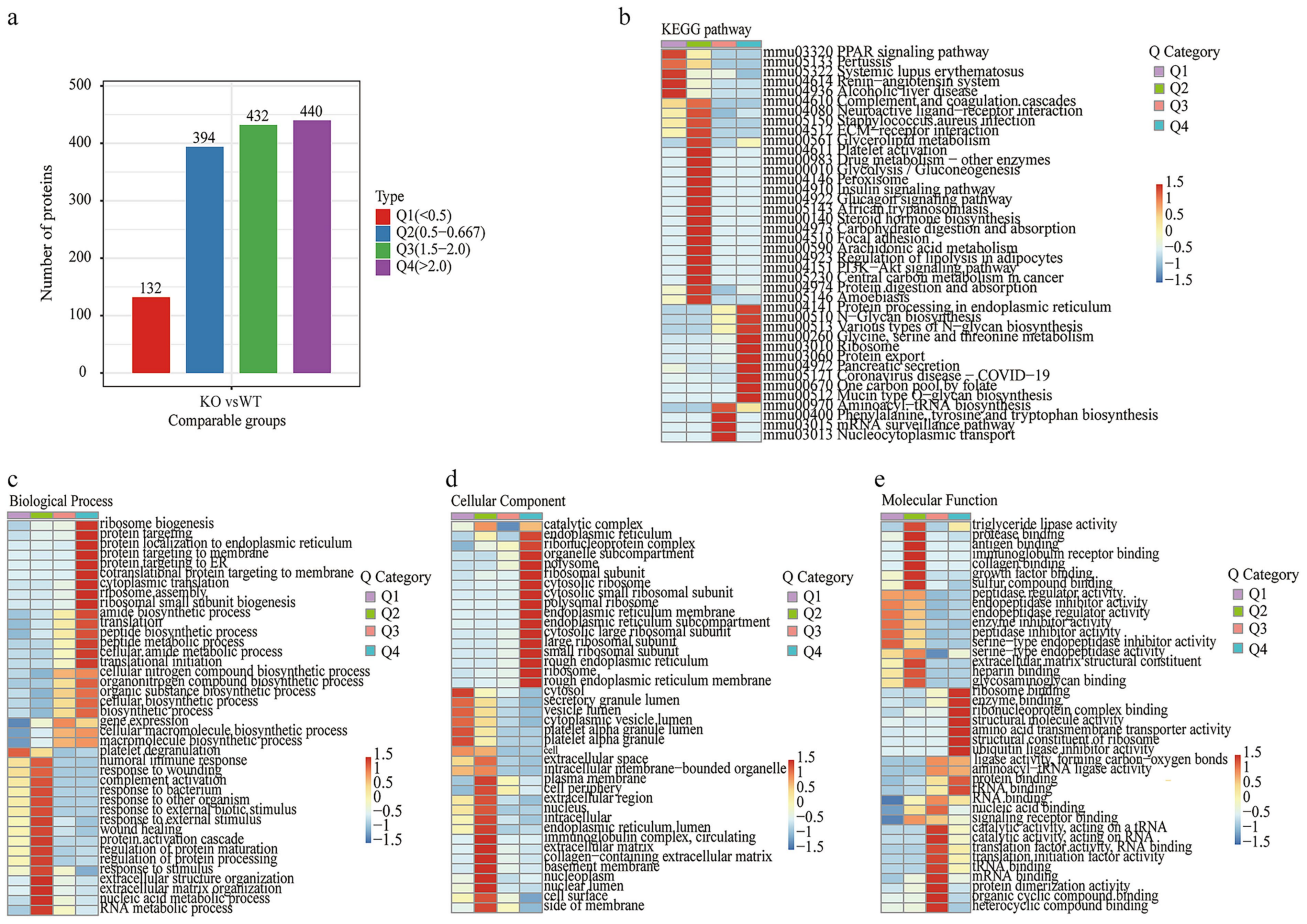
Clustering and enrichment analysis of the quantitative proteomics data. **(a)** The bar chart of differentially expressed proteins was further divided into four parts (Q1–Q4) according to the fold change. The color blocks corresponding to functional descriptions enriched for different Q groups and differentially expressed proteins indicate the strength and degree of enrichment. Red represents strong enrichment, whereas blue represents weak enrichment. **(b)** Kyoto Encyclopedia of Genes and Genomes (KEGG) pathway analysis after clustering. After clustering, the enriched genes were separated into **(c)** biological process (BP), **(d)** cellular component (CC), and **(e)** molecular function (MF) categories. The horizontal axis shows different Q groups, and the vertical axis shows the description of the enriched GO or KEGG pathways. Red indicates that the protein is strongly enriched, whereas blue indicates weak enrichment.

To better understand the changes in adipose tissue in VDRKO mice, we investigated the significant signaling pathways after cluster analysis. As shown in Figure 5a, the color bar on the right side of the bar chart displays the clustering group of the rows, which are divided into four clusters as presented by the four colors: Q1 (132 and red; fold change (FC) <0.5), Q2 (394 and blue; FC = 0.6–0.667), Q3 (432 and FC = 1.5–2.0), and Q4 (440 and purple; FC >2.0). Furthermore, pathway enrichment analysis was performed via the GO and KEGG databases. As shown in the heatmaps, the PPAR pathway, systemic lupus erythematosus, the renin–angiotensin system, and alcoholic liver disease pathways were enriched mainly in response to Q1. The Q2 group of proteins was enriched in neuroactive ligand–receptor interactions, extracellular matrix–receptor interactions, glycolysis–gluconeogenesis, and regulation of lipolysis in adipocytes. There are aminoacyl−tRNA biosynthesis; phenylalanine, tyrosine, and tryptophan biosynthesis; mRNA surveillance; and nucleocytoplasmic transport pathways enriched in the Q3 group. The DEPs are involved primarily in biosynthesis pathways and are associated with endoplasmic reticulum protein processing, ribosomes, and protein export, as shown in Q4 (Figure 5b).

GO functional enrichment among the diverse groups was divided into three categories: CC, MF, and BP (Figures 5c–e, respectively). This functional enrichment revealed many BP categories that were enriched for categories involved in biogenesis and the immune response, including ribosome biogenesis, protein localization to the endoplasmic reticulum, cytoplasmic translation, ribosome assembly, the humoral immune response, response to wounding, complement activation, the protein activation cascade, and RNA metabolism (Figure 5c). For CCs, the proteins of different groups were largely classified into the endoplasmic reticulum, ribonucleoprotein complex, organelle subcompartment, polysome, plasma membrane, cell periphery, extracellular region, nucleus, and intracellular regions (Figure 5d). The molecular function enrichment (Figure 5e) indicated that triglyceride lipase activity, protease binding, immunoglobulin receptor binding, ribosome binding, enzyme binding, ribonucleoprotein complex binding, and structural molecule activity were markedly changed.

**Figure 6:**
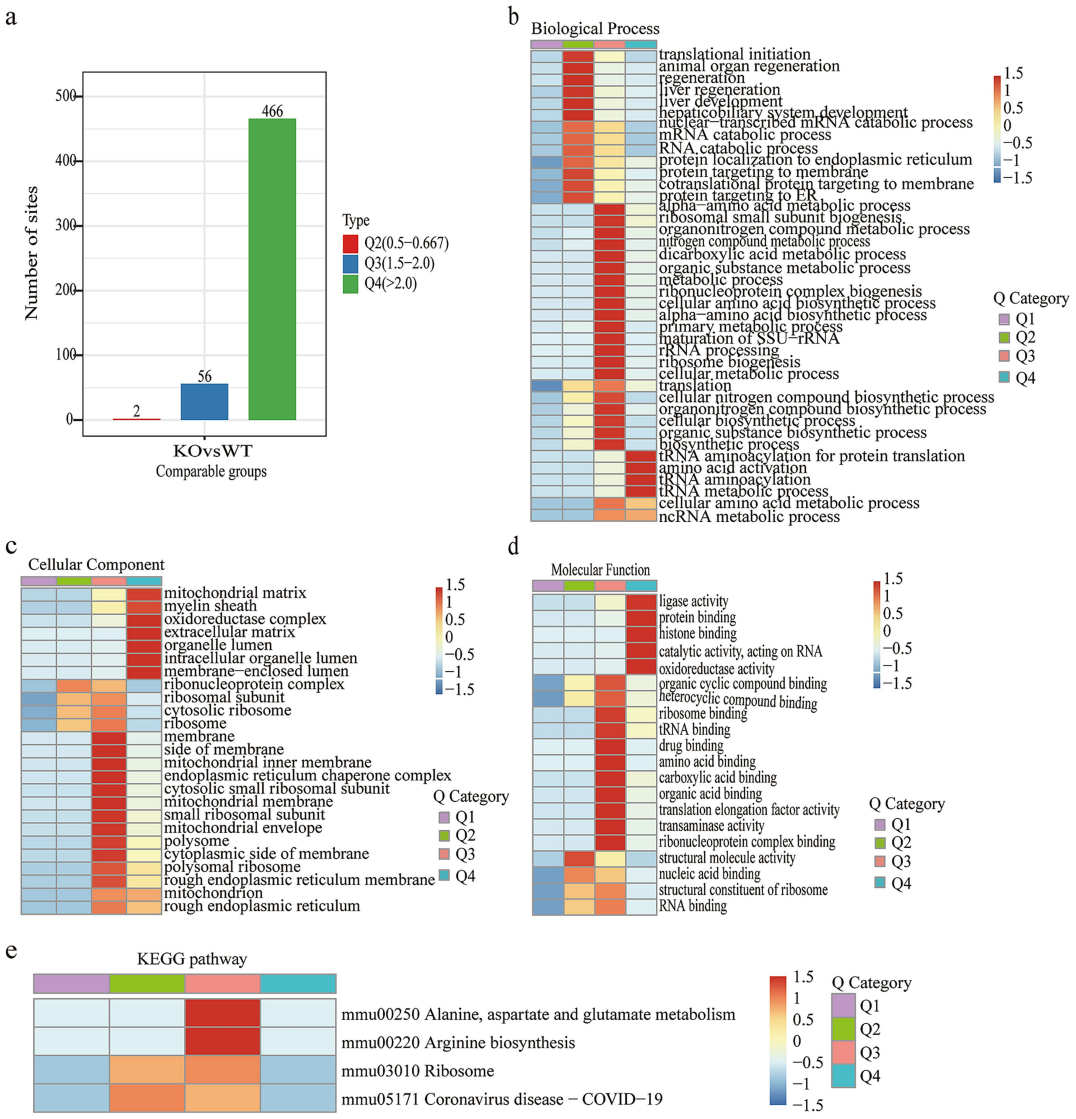
Clustering analysis of succinylated proteins. **(a)** Groups of succinylated differentially expressed proteins. **(b–d)** Gene Ontology (GO) classification after clustering. **(e)** Kyoto Encyclopedia of Genes and Genomes (KEGG) pathway analysis after clustering.

We also performed GO and KEGG analyses of the succinylome protein data on the basis of the fold change (FC) (Figure 6a). The GO terms for the succinylome are shown in Figures 6b–d. KEGG analysis revealed that alanine, aspartate, and glutamate metabolism; arginine biosynthesis; ribosomes; and coronavirus disease-COVID-19 were significantly enriched (Figure 6e).

### 5.4 Protein interaction networks identify key functional modules in VDRKO adipose tissue proteome and succinylome

**Figure 7:**
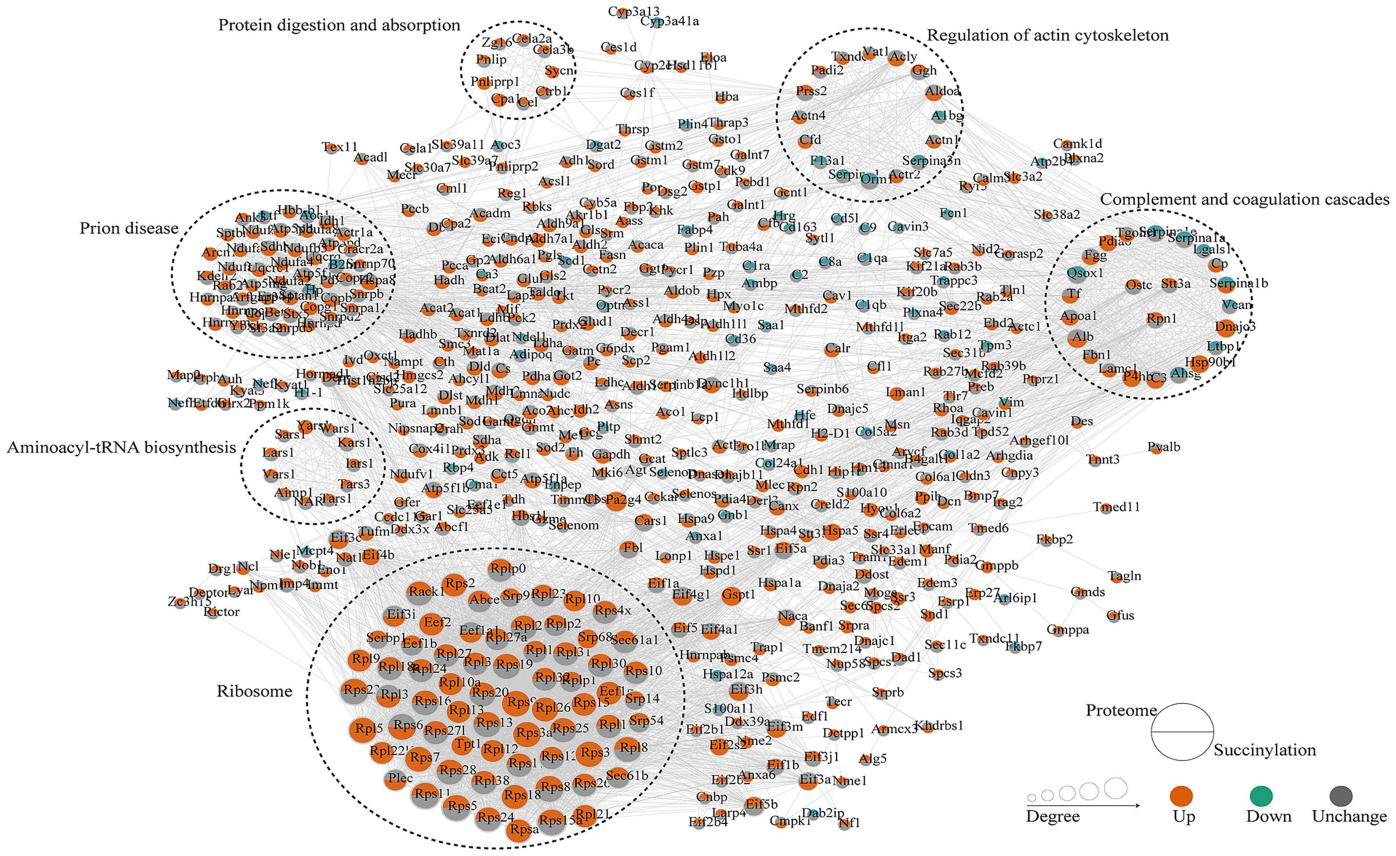
Protein–protein interaction (PPI) networks of the proteome and succinylome were constructed via the STRING database and visualized via Cytoscape (v3.8.2). The upper half of the circle represents the proteome, whereas the lower half represents succinylation. The node color is related to the change, and the size of the nodes is positively related to their betweenness. Red represents upregulation, green represents downregulation, and gray represents no change.

**Figure 8:**
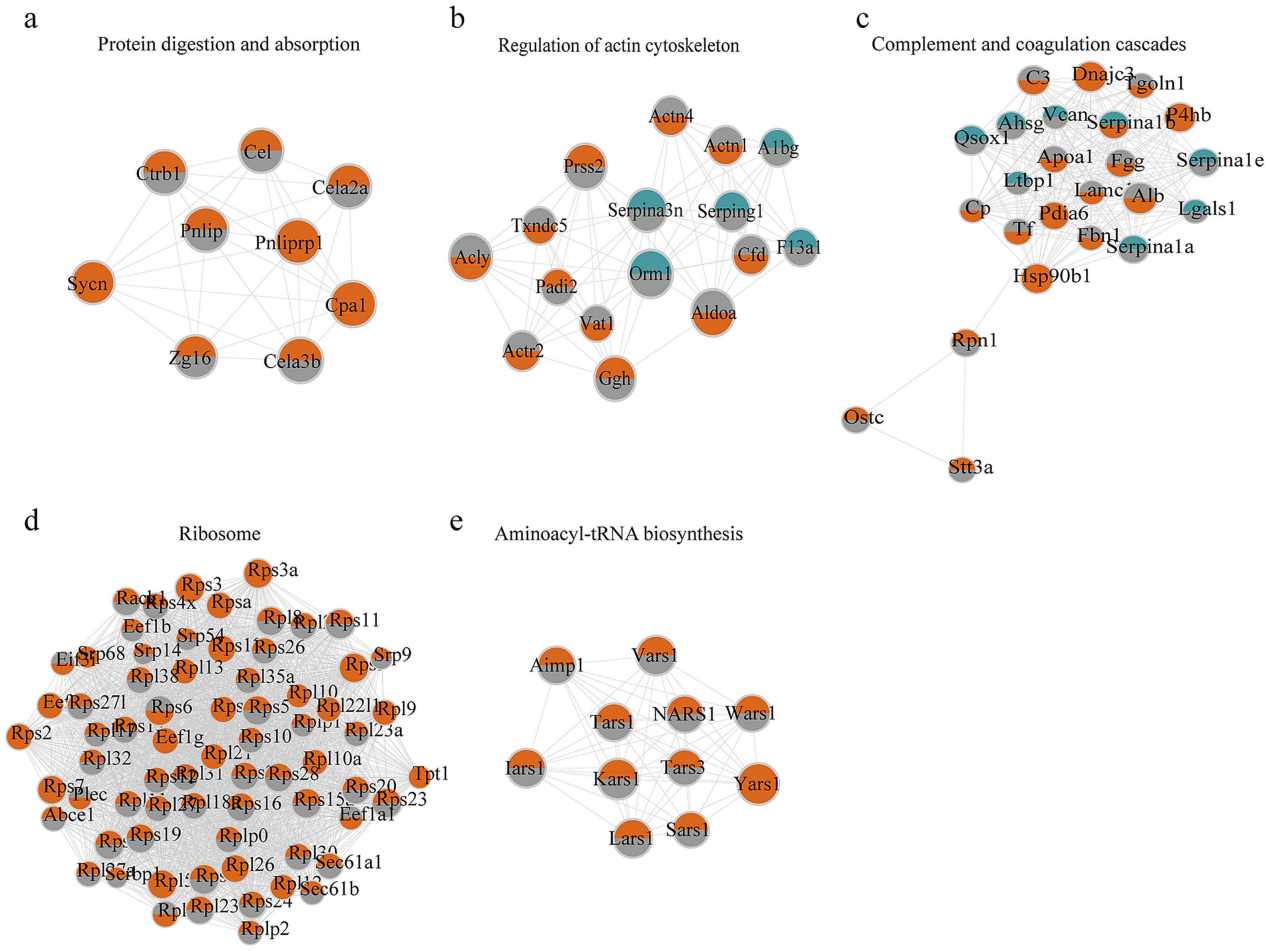
Top five enriched clusters of protein–protein interactions (PPIs) according to the proteomics and succinylomics results as visualized via Cytoscape MCOD. The size of the circle indicates the degree of correlation. Red represents up-regulation, green represents down-regulation, and gray represents no change.

Multidimensional omics data provide an opportunity to observe the proteome succinylome relationship of adipose tissue. STRING datasets—a search tool for interacting genes or proteins—were selected to visualize protein–protein interactions and were enriched in this dataset, thereby eliminating external molecules. The top six clusters for proteomic and succinylome characterization are shown in Figures 7, 8, respectively. There was an overlap between the functional analysis of differentially expressed succinylated proteins and proteins, revealing a remarkable overlap between the WT and VDRKO datasets (Figure 7). Our proteomic and succinylome data revealed an integrated landscape of VDRKO adipose tissues.

**Figure 9:**
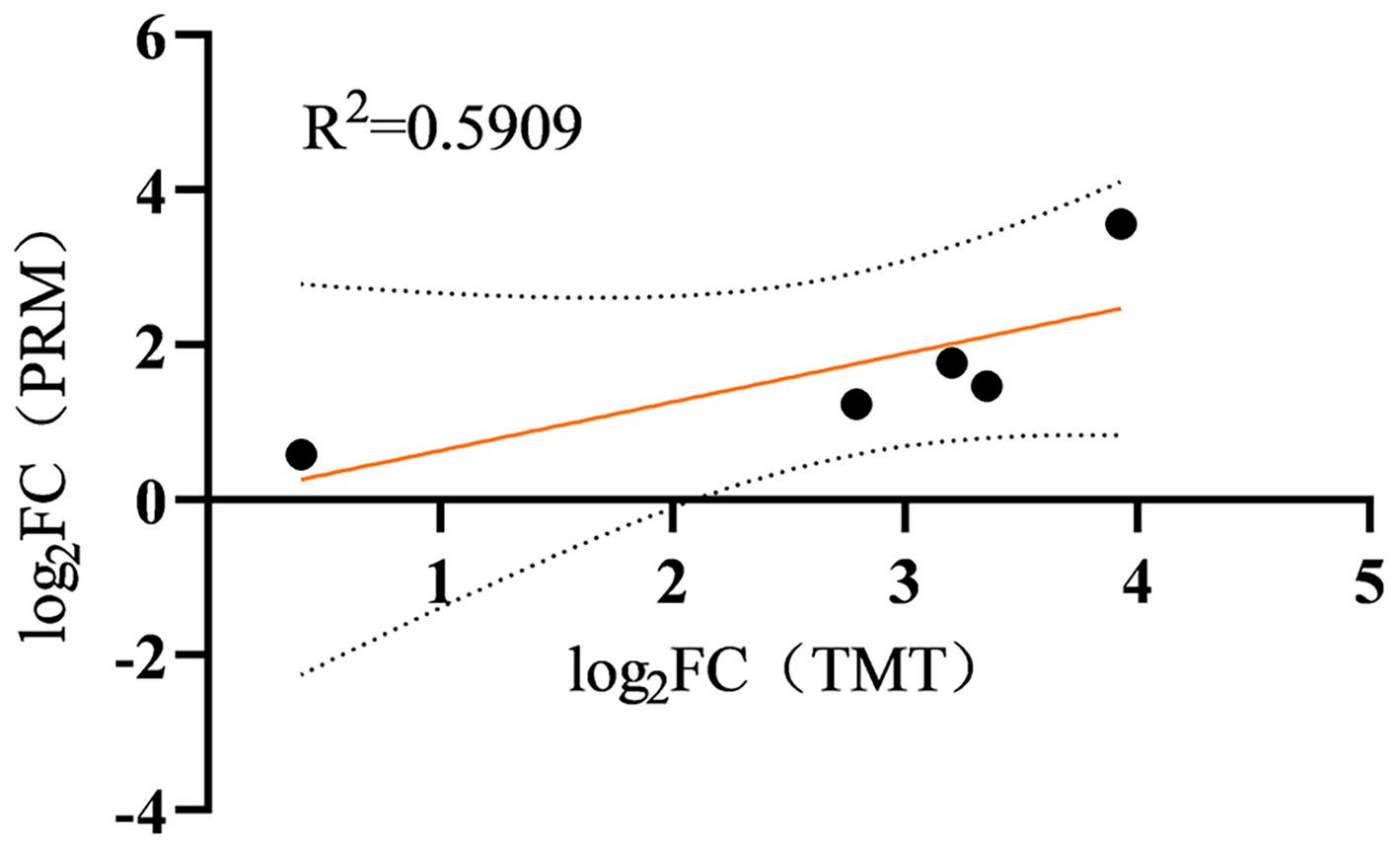
Changes in the five selected differentially expressed proteins (DEPs) through tandem mass tag (TMT) and parallel reaction monitoring (PRM) analysis. The selected DEPs, including pancreatic alpha-amylase (Amy2), proliferation-associated protein 2G4 (Pa2g4), 40S ribosomal protein S18 (Rps18), 40S ribosomal protein S3a (Rps3a), serine protease inhibitor A3N (Serpina3n).

To corroborate the TMT discovery set, five DEPs (differentially expressed proteins) were subjected to targeted quantification via PRM (Supplementary Table 1). The resulting abundance trajectories mirrored those detected in the TMT experiment (Figure 9), yielding an *R*^2^ of 0.5909, and thereby endorsing the reliability of the initial TMT measurements. PRM quantification was performed on 5 of the 15 selected target proteins. The results for the five proteins are shown in Table 1.

**Table 1:** Verification of the levels of differentially expressed proteins via PRM.

Protein accession	Protein description	Gene name	K/C ratio PRM	K/C ratio PRO
P00688	Pancreatic alpha-amylase	Amy2	3.56	3.93
P50580	Proliferation-associated protein 2G4	Pa2g4	1.77	3.2
P62270	40S ribosomal protein S18	Rps18	1.47	3.35
P97351	40S ribosomal protein S3a	Rps3a	1.24	2.79
Q91WP6	Serine protease inhibitor A3N	Serpina3n	0.59	0.4

PRM was quantified using the peak area. K/C ratio PRM: peak area distribution of the VDRKO/WT mouse ratio in the PRM analysis. K/C ratio PRO: peak area distribution of the VDRKO/WT mouse ratio according to proteomic analysis.

## 6 Discussion

To elucidate the effects of VDRKO on adipose tissue at the molecular level, we employed an integrated proteomic approach utilizing TMT labeling coupled with High-Performance Liquid Chromatography and liquid chromatography-tandem mass spectrometry (Figure 1). Our mass spectrometry data demonstrated excellent quality, with minimal average mass errors in peptide identification and appropriate peptide length distribution (8–20 amino acids), confirming the reliability of our proteomic profiles (Figures 1b,c). A key strength of our experimental design is the detailed documentation of nutritional composition, including macro-nutrients and vitamin D levels (4.99 × 10^3^ IU/kg), ensuring full transparency regarding dietary conditions that may influence adipose tissue metabolism.

These integrated proteomic and succinylomic analyses reveal that VDR deletion profoundly reprograms the adipose tissue landscape, providing molecular insights into the lean phenotype of VDRKO mice. Consistent with previous reports, VDRKO mice exhibited significantly lower body weights compared to WT controls, characterized by increased UCP1 expression and elevated energy expenditure (23, 25, 26). These phenotypic changes suggest substantial alterations in energy homeostasis and insulin sensitivity, potentially mediated through reduced adipose inflammation in the VDR-deficient state. Taken together, and within the documented nutritional context, our findings position reduced adipose inflammation as a potential central node linking VDR deletion to improved systemic energy homeostasis and insulin sensitivity. Building upon these phenotypic observations, our deep proteomic profiling quantified widespread protein expression changes, while subsequent succinylome analysis revealed extensive lysine succinylation modifications, particularly affecting mitochondrial and cytoplasmic proteins (12, 27, 28). Comprehensive functional annotation of the identified proteins revealed substantial alterations in catalytic and structural molecular activities in VDRKO adipose tissue (Figure 3). Protein–protein interaction network analysis further identified six significantly enriched clusters: ribosome, prion disease, complement and coagulation cascades, protein digestion and absorption, regulation of actin cytoskeleton, and aminoacyl-tRNA biosynthesis pathways (Figures 3–8). We interpret these pathway enrichments cautiously, recognizing they derive primarily from database annotations and require further experimental validation.

The proteomic signature indicated a substantial rewiring of metabolic pathways. Notably, ribosomal proteins were significantly upregulated in VDRKO adipose tissue, with PRM verification confirming increased expression of Rps3a and Rps18. These findings suggest potential alterations in translational regulation that may contribute to the metabolic adaptations observed in VDRKO mice (29, 30). Concurrently, we observed downregulation of complement and coagulation cascades, as well as protein digestion and absorption pathways, with collagen proteins such as Col24a1 emerging as key regulators (31–33). KEGG analysis further revealed downregulation of protein digestion and absorption pathways in VDRKO mice (Figure 4), with collagen proteins (Col24a1, Col6a4, Col1a2, and Col5a2) identified as key regulatory factors. These changes in extracellular matrix components may influence adipose tissue expansion and insulin sensitivity, potentially contributing to the improved metabolic profile of VDRKO mice.

Subcellular localization analysis revealed that mitochondrial proteins showed the most pronounced increase in succinylation (31), consistent with the central role of mitochondria in energy metabolism and the generation of succinyl-CoA, the essential substrate for this modification. This mitochondrial predominance is mechanistically justified by the proximity to succinyl-CoA production sites and the abundance of metabolic enzymes in this compartment. The functional impact of succinylation on metabolic enzymes is potentially profound, as this modification can alter enzyme activity, stability, and protein interactions. Mechanistically, these changes may be driven by altered activity of enzymes regulating the succinylome, such as the desuccinylase SIRT5 or metabolic enzymes like succinate dehydrogenase (SDH), which influence succinyl-CoA pools (32–37). The enrichment of succinylation in metabolic pathways including alanine, aspartate, and glutamate metabolism and arginine biosynthesis provides a direct mechanistic link between VDR deletion and the observed metabolic phenotype (38–40). Specifically, succinylation of key metabolic enzymes in these pathways could directly modulate their activity, thereby influencing energy homeostasis and adipocyte function. This widespread rewiring of the succinylome represents a novel mechanism through which VDR deletion may drive metabolic reprogramming in adipose tissue. Thus, the extensive succinylation of mitochondrial metabolic enzymes emerges as a pivotal post-translational mechanism that directly links VDR ablation to the enhanced energy expenditure and lean phenotype observed in VDRKO mice.

Pathway analysis demonstrated significant alterations in multiple biological processes (Figure 4). Upregulated proteins were predominantly associated with ribosome, COVID-19, and endoplasmic reticulum protein processing pathways (Figure 4e), while downregulated proteins were enriched in complement and coagulation cascades, systemic lupus erythematosus, and protein digestion and absorption pathways (Figure 4f). We specifically frame the observed COVID-19 pathway components within the context of general immune and inflammatory signaling mechanisms rather than viral susceptibility per se. Specifically, the complement and coagulation cascades, which were enriched in lean WT mice, showed significant downregulation in VDRKO mice (Figures 4, 5b), aligning with transcriptomic patterns observed in lean versus obese canine models (41, 42). Similarly, changes in systemic lupus erythematosus pathway components reflect alterations in general immune regulation rather than specific autoimmune pathogenesis. The reduced activity of the systemic lupus erythematosus pathway in VDRKO adipose tissue (Figure 4f) corroborates previous epidemiological links between vitamin D deficiency and autoimmune disease incidence (43).

Our succinylome analysis provides mechanistic insight into the metabolic phenotype of VDRKO mice. The alterations in endoplasmic reticulum protein processing pathways align with previous reports of vitamin D3’s role in regulating ER function (44). The extensive succinylation modifications observed, particularly affecting mitochondrial proteins, directly impact key metabolic enzymes involved in energy homeostasis. We also detected substantial succinylation modifications in cytoplasmic compartments, suggesting a broader regulatory role for succinylation beyond mitochondrial metabolism in VDR-deficient states. Future studies should directly assess the role of specific succinylome regulators in VDR-deficient adipose tissue.

Beyond the alterations in organelle function, the distinction between our VDR knockout model and physiological vitamin D deficiency warrants consideration (45, 48). While both impair vitamin D signaling, fundamental mechanistic differences likely yield divergent proteomic profiles (46). VDRKO causes complete, cell-autonomous receptor loss, whereas dietary deficiency represents ligand deprivation with preserved receptor functionality. Although both conditions share phenotypes like leanness and UCP1 upregulation, our integrated succinylome analysis reveals extensive, unique post-translational modifications in metabolic enzymes in VDRKO mice (47). This suggests that constitutive receptor absence drives a more profound metabolic reprogramming than ligand deficiency alone. Thus, while VDRKO is invaluable for identifying cell-autonomous VDR functions, our findings represent the severe end of the vitamin D signaling deficiency spectrum and may not be fully translatable to human dietary deficiency.

Several methodological limitations should be considered together in interpreting our results. The relatively small sample size (*n* = 3 per group) may affect statistical power, and the exclusive use of male mice limits generalizability to both sexes, consequently, findings require validation in larger, hypothesis-driven cohorts. Because female mice exhibit oestrous-cycle-driven metabolic fluctuations, the present findings require validation in females. The dietary composition, while documented for transparency, was not experimentally manipulated, which could influence both vitamin D status and adipose tissue metabolism. Additionally, while we validated several key proteins through PRM, comprehensive validation of all identified changes was not feasible. Furthermore, the absence of histological analysis of white and brown adipose tissue precludes a direct correlation between the observed molecular alterations and tissue-level morphological changes, such as adipocyte size, crown-like structures, or mitochondrial density. Similarly, we did not assess the functional consequence of VDR deletion on thermogenic capacity through cold tolerance tests, which would provide direct physiological evidence for altered adipose tissue energy expenditure. Our translational statements are therefore intentionally moderate and hypothesis-driven, focusing on the mechanistic insights provided by this comprehensive molecular profiling rather than implying direct clinical applicability.

Despite these limitations, our study provides valuable insights into the molecular consequences of VDR deletion in adipose tissue. The extensive rewiring of both the proteome and succinylome suggests that VDR signaling plays a crucial role in maintaining adipose tissue homeostasis. The VDRKO mouse model, while not fully recapitulating human vitamin D deficiency states, offers a valuable system for studying cell-autonomous effects of VDR signaling.

Future studies should focus on validating the functional significance of specific protein changes and succinylation modifications identified in this study. Particularly, the role of ribosomal proteins and metabolic enzymes in mediating the VDRKO phenotype deserves further exploration. Additionally, investigating how these molecular changes translate to alterations in insulin sensitivity, inflammatory status, and overall metabolic function will be crucial for understanding the physiological relevance of our findings.

In conclusion, our integrated multi-omics approach provides a comprehensive characterization of the proteomic and succinylomic landscape in VDRKO adipose tissue. The integration of advanced mass spectrometry techniques with systematic bioinformatic analyses has identified distinct pathway alterations and subcellular distribution patterns that advance our understanding of VDR’s role in metabolic regulation. The extensive changes in both protein expression and succinylation modification highlight the complex regulatory networks controlled by vitamin D signaling in adipose tissue, offering new perspectives for understanding metabolic diseases and potential therapeutic strategies. These findings establish a foundation for future mechanistic studies investigating VDR-mediated processes in adipose tissue biology and related metabolic disorders.

## Data Availability

The mass spectrometry proteomics data have been deposited to the ProteomeXchange Consortium (https://proteomecentral.proteomexchange.org) via the iProX partner repository with the dataset identifier PXD074869.

## Footnotes

1
http://www.ebi.ac.uk/GOA/

2
http://www.genome.jp/tools/kaas/

3
https://www.kegg.jp/kegg/kegg1b.html

4
https://string-db.org/

## References

[1] Çizmecioğlu FM Etiler N Görmüş U Hamzaoğlu O Hatun Ş. Hypovitaminosis D in obese and overweight schoolchildren. J Clin Res Pediatr Endocrinol. (2008) 1:89–96. doi: 10.4008/jcrpe.v1i2.43, 21318069 PMC3005643

[2] Miñambres I Sánchez-Quesada JL Vinagre I Sánchez-Hernández J de Leiva A Pérez A. Hypovitaminosis D in type 2 diabetes: relation with features of the metabolic syndrome and glycemic control. Endocr Res. (2015) 40:160–5. doi: 10.3109/07435800.2014.982326, 25536005

[3] Valer-Martinez A Martinez JA Sayon-Orea C Galvano F Grosso G Bes-Rastrollo M. Vitamin D and cardio-metabolic risk factors in overweight adults: an overview of the evidence. Curr Pharm Des. (2019) 25:2407–20. doi: 10.2174/1381612825666190722103919, 31333117

[4] Lu S Cao ZB. Interplay between vitamin D and adipose tissue: implications for adipogenesis and adipose tissue function. Nutrients. (2023) 15:4832. doi: 10.3390/nu15224832 38004226 PMC10675652

[5] Pakosiński M Żyła M Kamieniak A Kluz N Gil-Kulik P. Vitamin D receptor polymorphisms and immunological effects of vitamin D in Hashimoto’s thyroiditis. Int J Mol Sci. (2025) 26:10576. doi: 10.3390/ijms262110576 41226614 PMC12609546

[6] Li A Zhang W Zhang H Yi B. Vitamin D/vitamin D receptor, autophagy and inflammation relevant diseases. Zhong Nan Da Xue Xue Bao Yi Xue Ban. (2017) 42:979–85. doi: 10.11817/j.issn.1672-7347.2017.08.017 28872092

[7] Wong KE Szeto FL Zhang W Ye H Kong J Zhang Z . Involvement of the vitamin D receptor in energy metabolism: regulation of uncoupling proteins. Am J Physiol Endocrinol Metab. (2009) 296:E820–8. doi: 10.1152/ajpendo.90763.2008, 19176352 PMC2670625

[8] Moscalu A Stupalkowska W Zhao L Li Y Roberts CF Chen Y . (2025). Vitamin D receptor is necessary for metabolic health after sleeve gastrectomy. *bioRxiv*. Available online at: 10.1101/2025.10.24.684160. [Epub ahead of preprint]

[9] Narvaez CJ Matthews D Broun E Chan M Welsh J. Lean phenotype and resistance to diet-induced obesity in vitamin D receptor knockout mice correlates with induction of uncoupling protein-1 in white adipose tissue. Endocrinology. (2009) 150:651–61. doi: 10.1210/en.2008-1118, 18845643 PMC2646525

[10] Ji L Gupta M Feldman BJ. Vitamin D regulates fatty acid composition in subcutaneous adipose tissue through Elovl3. Endocrinology. (2016) 157:91–7. doi: 10.1210/en.2015-1674, 26488808 PMC4701879

[11] Lee MJ. Vitamin D enhancement of adipose biology: implications on obesity-associated Cardiometabolic diseases. Nutrients. (2025) 17:586. doi: 10.3390/nu17030586, 39940444 PMC11820181

[12] Su H Lou Y Fu Y Wang C Liu Y Li D . Involvement of the vitamin D receptor in energy metabolism revealed by profiling of lysine succinylome of white adipose tissue. Sci Rep. (2017) 7:14132. doi: 10.1038/s41598-017-14477-8, 29074956 PMC5658373

[13] Kershaw EE Flier JS. Adipose tissue as an endocrine organ. J Clin Endocrinol Metab. (2004) 89:2548–56. doi: 10.1210/jc.2004-0395, 15181022

[14] McGown C Birerdinc A Younossi ZM. Adipose tissue as an endocrine organ. Clin Liver Dis. (2014) 18:41–58. doi: 10.1016/j.cld.2013.09.012 24274864

[15] Wang H Yang L Liu M Luo J. Protein post-translational modifications in the regulation of cancer hallmarks. Cancer Gene Ther. (2023) 30:529–47. doi: 10.1038/s41417-022-00464-3 35393571

[16] Jeon SM Shin EA. Exploring vitamin D metabolism and function in cancer. Exp Mol Med. (2018) 50:1–14. doi: 10.1038/s12276-018-0038-9, 29657326 PMC5938036

[17] Wang Y Tong M. Protein posttranslational modification in stemness remodeling and its emerging role as a novel therapeutic target in gastrointestinal cancers. Int J Mol Sci. (2023) 24:9173. doi: 10.3390/ijms24119173, 37298124 PMC10252960

[18] He S Wang C Li R Liu Y Zhang H Wang Y . The role of succinylation-mediated metabolic reprogramming in tumor progression. Mol Biol Rep. (2025) 52:954. doi: 10.1007/s11033-025-11061-6, 41003805

[19] Wang G Meyer JG Cai W Softic S Li ME Verdin E . Regulation of UCP1 and mitochondrial metabolism in brown adipose tissue by reversible succinylation. Mol Cell. (2019) 74:844–857.e7. doi: 10.1016/j.molcel.2019.03.021, 31000437 PMC6525068

[20] Abbas MA. Physiological functions of vitamin D in adipose tissue. J Steroid Biochem Mol Biol. (2017) 165:369–81. doi: 10.1016/j.jsbmb.2016.08.004, 27520301

[21] Moreno-Santos I Castellano-Castillo D Lara MF Fernandez-Garcia JC Tinahones FJ Macias-Gonzalez M. IGFBP-3 interacts with the vitamin D receptor in insulin signaling associated with obesity in visceral adipose tissue. Int J Mol Sci. (2017) 18:2349. doi: 10.3390/ijms18112349, 29112142 PMC5713318

[22] Narvaez CJ Simmons KM Brunton J Salinero A Chittur SV Welsh JE. Induction of STEAP4 correlates with 1,25-dihydroxyvitamin D3 stimulation of adipogenesis in mesenchymal progenitor cells derived from human adipose tissue. J Cell Physiol. (2013) 228:2024–36. doi: 10.1002/jcp.24371, 23553608

[23] Bouillon R Carmeliet G Lieben L Watanabe M Perino A Auwerx J . Vitamin D and energy homeostasis: of mice and men. Nat Rev Endocrinol. (2014) 10:79–87. doi: 10.1038/nrendo.2013.226, 24247221

[24] Li YC Pirro AE Amling M Delling G Baron R Bronson R . Targeted ablation of the vitamin D receptor: an animal model of vitamin D-dependent rickets type II with alopecia. Proc Natl Acad Sci USA. (1997) 94:9831–5. doi: 10.1073/pnas.94.18.9831, 9275211 PMC23277

[25] Su H Liu N Zhang Y Kong J. Vitamin D/VDR regulates peripheral energy homeostasis via central renin-angiotensin system. J Adv Res. (2021) 33:69–80. doi: 10.1016/j.jare.2021.01.011, 34603779 PMC8463910

[26] Lau SL Stokes RA Ng B Cheng K Clifton-Bligh R Gunton JE. Metabolic changes in vitamin D receptor knockout mice. PLoS One. (2022) 17:e0267573. doi: 10.1371/journal.pone.0267573, 35714079 PMC9205491

[27] Hu B Gong H Nie L Zhang J Li Y Liu D . Lysine succinylation precisely controls normal erythropoiesis. Haematologica. (2025) 110:397–413. doi: 10.3324/haematol.2024.285752, 39415677 PMC11788629

[28] Zamanian Azodi M Peyvandi H Rostami-Nejad M Safaei A Rostami K Vafaee R . Protein-protein interaction network of celiac disease. Gastroenterol Hepatol Bed Bench. (2016) 9:268–77. doi: 10.15171/ghbb.2016.36 27895852 PMC5118851

[29] Kim JY Atanassov I Dethloff F Kroczek L Langer T. Time-resolved proteomic analyses of senescence highlight metabolic rewiring of mitochondria. Life Sci Alliance. (2023) 6:e202302127. doi: 10.26508/lsa.202302127, 37321846 PMC10272782

[30] Ren W Hou X Wang Y Badgery W Li X Ding Y . Overgrazing induces alterations in the hepatic proteome of sheep (*Ovis aries*): an iTRAQ-based quantitative proteomic analysis. Proteome Sci. (2016) 15:2. doi: 10.1186/s12953-016-0111-z, 28149202 PMC5267464

[31] Passos JRS Guerreiro DD Otávio KS dos Santos‐Neto PC Souza‐Neves M Cuadro F . How *in vitro* maturation changes the proteome of ovine cumulus-oocyte complexes? Mol Reprod Dev. (2022) 89:459–70. doi: 10.1002/mrd.23638, 35901249

[32] Xiao H Xin W Sun LM Li SS Zhang T Ding XY. Comprehensive proteomic profiling of aqueous humor proteins in proliferative diabetic retinopathy. Transl Vis Sci Technol. (2021) 10:3. doi: 10.1167/tvst.10.6.3, 34111250 PMC8107506

[33] Li N Zhao X You S. Identification of key regulators of pancreatic ductal adenocarcinoma using bioinformatics analysis of microarray data. Medicine. (2019) 98:e14074. doi: 10.1097/MD.0000000000014074, 30633213 PMC6336631

[34] Witten AJ Ejendal KFK Gengelbach LM Traore MA Wang X Umulis DM . Fluorescent imaging of protein myristoylation during cellular differentiation and development. J Lipid Res. (2017) 58:2061–70. doi: 10.1194/jlr.D074070, 28754825 PMC5625117

[35] Bai F Ma Y Liu Q. Succinylation as a novel mode of energy metabolism regulation during atrial fibrillation. Med Hypotheses. (2018) 121:54–5. doi: 10.1016/j.mehy.2018.09.018, 30396491

[36] Li F He X Ye D Lin Y Hongxiu Y Yao C . NADP^+^-IDH mutations promote hypersuccinylation that impairs mitochondria respiration and induces apoptosis resistance. Mol Cell. (2015) 60:661–75. doi: 10.1016/j.molcel.2015.10.017, 26585387

[37] Liu L Wang Q Zhao B Wu Q Wang P. Exogenous nicotinamide adenine dinucleotide administration alleviates ischemia/reperfusion-induced oxidative injury in isolated rat hearts via Sirt5-SDH-succinate pathway. Eur J Pharmacol. (2019) 858:172520. doi: 10.1016/j.ejphar.2019.172520, 31278893

[38] Tao T Kobelski MM Saini V Demay MB. Adipose-specific VDR deletion leads to hepatic steatosis in female mice fed a low-fat diet. Endocrinology. (2022) 163:bqab249. doi: 10.1210/endocr/bqab249, 34878523 PMC10061053

[39] Lou Y Dong C Jiang Q He Z Yang S. Protein succinylation mechanisms and potential targeted therapies in urinary disease. Cell Signal. (2025) 131:111744. doi: 10.1016/j.cellsig.2025.111744, 40090556

[40] Lian J Liu W Hu Q Zhang X. Succinylation modification: a potential therapeutic target in stroke. Neural Regen Res. (2024) 19:781–7. doi: 10.4103/1673-5374.382229, 37843212 PMC10664134

[41] Grant RW Vester Boler BM Ridge TK Graves TK Swanson KS. Subcutaneous and gonadal adipose tissue transcriptome differences in lean and obese female dogs. Anim Genet. (2013) 44:728–35. doi: 10.1111/age.12060, 23713485

[42] Park H Imoto S Miyano S. Gene behaviors-based network enrichment analysis and its application to reveal immune disease pathways enriched with COVID-19 severity-specific gene networks. Bioinformatics. (2025) 41:btaf378. doi: 10.1093/bioinformatics/btaf378, 40580453 PMC12270264

[43] Yamamoto E Jorgensen TN. Immunological effects of vitamin D and their relations to autoimmunity. J Autoimmun. (2019) 100:7–16. doi: 10.1016/j.jaut.2019.03.002 30853311

[44] Song D Liu H Wu J Gao X Hao J Fan D. Insights into the role of ERp57 in cancer. J Cancer. (2021) 2:2456–64. doi: 10.7150/jca.48707 PMC7974888 33758622

[45] Liu S Wang X Xianyong B Lin Z Li E Shi Q . Impact of dietary vitamin D3 supplementation on growth, molting, antioxidant capability, and immunity of juvenile Chinese mitten crabs (*Eriocheir sinensis*) by metabolites and vitamin D receptor. J Agric Food Chem. (2021) 69:12794–806. doi: 10.1021/acs.jafc.1c04204 34677964

[46] Feigerlova E Demarquet L Melhem H Ghemrawi R Battaglia-Hsu SF Ewu E . Methyl donor deficiency impairs bone development via peroxisome proliferator-activated receptor-γ coactivator-1α-dependent vitamin D receptor pathway. FASEB J. (2016) 30:3598–612. doi: 10.1096/fj.201600332R, 27435264

[47] Hosoyama T Kawai-Takaishi M Iida H Yamamoto Y Nakamichi Y Watanabe T . Lack of vitamin D signalling in mesenchymal progenitors causes fatty infiltration in muscle. J Cachexia Sarcopenia Muscle. (2024) 15:907–18. doi: 10.1002/jcsm.13448 38533539 PMC11154772

[48] Demay MB. Physiological insights from the vitamin D receptor knockout mouse. Calcif Tissue Int. (2013) 92:99–105. doi: 10.1007/s00223-012-9633-2, 22903507 PMC3511627

